# Prospective study of oncologic outcomes after laparoscopic modified complete mesocolic excision for non-metastatic right colon cancer (PIONEER study): study protocol of a multicentre single-arm trial

**DOI:** 10.1186/s12885-020-07151-2

**Published:** 2020-07-14

**Authors:** Seung Yoon Yang, Min Jung Kim, Bong-Hyeon Kye, Yoon Dae Han, Min Soo Cho, Seung-Yong Jeong, Hyeon-Min Cho, Hyunki Kim, Gyeong Hoon Kang, Seung Ho Song, Jun Seok Park, Ji-Seon Kim, Soo Yeun Park, Jin Kim, Byung Soh Min

**Affiliations:** 1 Department of Surgery, Yonsei University College of Medicine, Severance Hospital, 50 Yonsei-ro, Seodaemun-ku, Seoul, 120-752 South Korea; 2 Department of Surgery, Seoul National University College of Medicine, Seoul, South Korea; 3 Department of Surgery, Catholic University of Korea School of Medicine, Seoul, South Korea; 4 Department of Pathology, Yonsei University College of Medicine, Seoul, South Korea; 5 Department of Pathology, Seoul National University College of Medicine, Seoul, South Korea; 6 Department of Surgery, School of Medicine, Kyungpook National University, Daegu, South Korea; 7 Department of Surgery, Korea University College of Medicine, 73 Goryeodae-ro, Seoul, South Korea

**Keywords:** Modified complete mesocolic excision, Laparoscopic surgery, Right-sided colon cancer, Oncologic outcomes

## Abstract

**Background:**

The introduction of complete mesocolic excision (CME) with central vascular ligation (CVL) for right-sided colon cancer has improved the oncologic outcomes. Recently, we have introduced a modified CME (mCME) procedure that keeps the same principles as the originally described CME but with a more tailored approach. Some retrospective studies have reported the favourable oncologic outcomes of laparoscopic mCME for right-sided colon cancer; however, no prospective multicentre study has yet been conducted.

**Methods:**

This study is a multi-institutional, prospective, single-arm study evaluating the oncologic outcomes of laparoscopic mCME for adenocarcinoma arising from the right side of the colon. A total of 250 patients will be recruited from five tertiary referral centres in South Korea. The primary outcome of this study is 3-year disease-free survival. Secondary outcome measures include 3-year overall survival, incidence of surgical complications, completeness of mCME, and distribution of metastatic lymph nodes. The quality of laparoscopic mCME will be assessed on the basis of photographs of the surgical specimen and the operation field after the completion of lymph node dissection.

**Discussion:**

This is a prospective multicentre study to evaluate the oncologic outcomes of laparoscopic mCME for right-sided colon cancer. To the best of our knowledge, this will be the first study to prospectively and objectively assess the quality of laparoscopic mCME. The results will provide more evidence about oncologic outcomes with respect to the quality of laparoscopic mCME in right-sided colon cancer.

**Trial registration:**

ClinicalTrials.gov ID: NCT03992599 (June 20, 2019). The posted information will be updated as needed to reflect protocol amendments and study progress.

## Background

With the introduction of complete mesocolic excision (CME) with central vascular ligation (CVL), the outcome of colon cancer surgery has significantly improved [1–4]. CME in colon cancer surgery is a concept analogous to total mesorectal excision in rectal cancer, which is based on sharp dissection along the embryological anatomical planes with sharp separation of the visceral fascia from the parietal plane, leading to a surgical specimen with an intact coverage [2, 5]. The CME technique involves oncologic resection with careful dissection of the mesocolon along the embryological planes, resulting in the complete mobilisation of the mesocolon covered by an intact visceral fascia layer containing all blood vessels, lymphatic vessels, and lymph nodes that may contain disseminated cancer cells [1, 4]. Moreover, the method of ligating the supplying vessels at their origin (CVL) and removing the entire mesocolon has a considerable effect on locoregional recurrence and improves oncologic outcomes [1, 3]. It is well known that excision of specimens with an intact mesocolon is associated with better survival rates than excision of specimens with a defective mesocolon [3, 4].

Although Hohenberger et al. first used the term CME with CVL [2], the concept is not necessarily new because many institutions have already accepted similar concepts. In particular, many Japanese surgeons would argue that they have been performing a similar procedure, known as D3 dissection [6]. Despite sharing similar concepts, the technical details may differ between CME with CVL and Japanese D3 dissection, as demonstrated by differences in the length of resected bowels and the area of the excised mesocolon, which seem to result from different definitions of an adequate resection margin [7].

In practice, CME for right-sided colon cancer seems to be more challenging because of the complexity and variability of the central vascular anatomy than with a left-sided disease. Moreover, the original description of CME for right-sided colon cancer indicates a very aggressive procedure including complete Kocherization and extensive extra-mesocolic lymph node dissection regardless of the tumour location and stage, which translates to a higher risk of serious postoperative complications, especially when performed laparoscopically [2, 8, 9].

Recently, we have introduced a modified CME (mCME) technique for right-sided colon cancer that keeps the same principles as the original CME procedure but with a more tailored approach according to the location and stage of the tumour. This tailored approach focuses on three main points: 1) achievement of an adequate radial margin, 2) tailored lymphadenectomy according to tumour location, and 3) selective extra-mesocolic lymph node dissection [10].

Thus far, a considerable number of studies have shown favourable oncologic outcomes and short-term outcomes with the CME, mCME, and Japanese D3 types of dissection compared with conventional surgery in right-sided colon cancer [3, 6, 10, 11]. However, to the best of our knowledge, no study has proven the oncologic safety of the laparoscopic approach with objective surgical quality assessment.

This is a multi-institutional, prospective, single-arm study designed to evaluate the oncologic outcomes after laparoscopic mCME for adenocarcinoma arising from the right side of the colon, which is defined as from the caecum up to the proximal half of the transverse colon. In this study, the participating surgeons are assumed to have overcome their learning curve and will be evaluated beforehand by independent experts. The surgical quality will be assessed on the basis of both, the resected surgical specimen and the operation field after specimen removal, using submitted photographs.

## Methods

### Study design

This is a multi-institutional, prospective, single-arm study. The duration of the study will be approximately 5 years (2 years of inclusion, 3 years of follow-up). Patients will be enrolled at five tertiary colorectal cancer centres in South Korea, including Yonsei Cancer Center, Seoul National University Hospital, St. Vincent’s Hospital, Kyungpook National University Chilgok Hospital, and Korea University Anam Hospital. A complete information leaflet will be provided to the patients during the first consultation, and informed consent will be obtained from them after screening. The preoperative, intraoperative, and postoperative periods will be in complete accordance with the usual care practices of the centres. The baseline demographics and conditions, as well as the perioperative details and the postoperative occurrences, will be recorded on a previously designed case report form. Data from each participating hospital will be collected at the PIONEER database. Data collection forms can be assessed after login on the website. Each hospital has access to their own dataset. The follow-up encompasses 13 postoperative consultations: 1 month, 3 months, and every 3 months thereafter until 36 months.

### Ethics approval

Before the enrolment of the first patient, approval for this study will be obtained from the institutional review board of each participating research centre (five tertiary hospitals) in South Korea. Written informed consent will be obtained from all patients for the acquisition and use of anonymised clinical data before they are recruited, and all investigators will conduct this study in accordance with the tenets of the Declaration of Helsinki. This study will be monitored by an independent data and safety monitoring committee.

### Study population

**Fig. 1:**
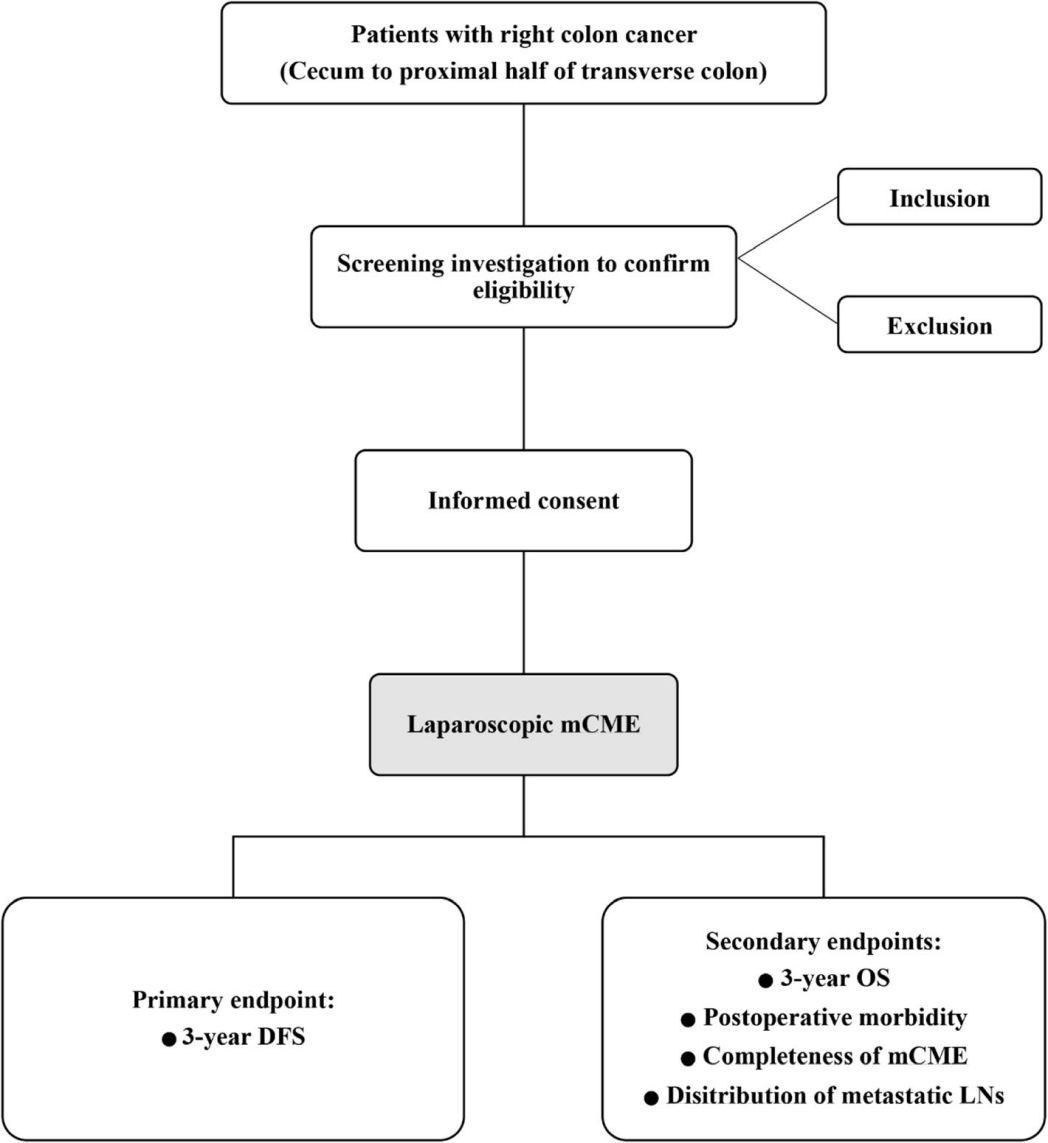
Flow chart of the study

Patients with adenocarcinoma arising from the right side of the colon who are indicated to undergo a laparoscopic mCME will be eligible for this study. The right side of the colon is defined as from caecum up to the proximal half of the transverse colon. Investigators from each institution will be responsible for the enrolment according to the inclusion/exclusion criteria and patient conditions. The flow of participant inclusion is schematically shown in Fig. 1.

### Inclusion criteria

- Qualitative diagnosis: a pathological diagnosis of adenocarcinoma, with the tumour located between the caecum and the proximal half of the transverse colon
- Suitability for curative surgery and age > 19 years
- American Society of Anesthesiologists physical status grade I-III
- No preoperative treatment
- Provision of written informed consent

### Exclusion criteria

- Need for an emergency operation because of conditions such as perforation or malignant colonic obstruction
- Preoperative imaging examination results showing distant metastasis
- Hereditary colon cancer
- History of any other malignant tumour in the recent 5 years, except for cervical carcinoma in situ that has been cured, basal cell carcinoma, or squamous cell carcinoma of the skin
- Simultaneous or simultaneous multiple primary colorectal cancer
- Pregnancy or breastfeeding in women
- Patients who are not suitable to undergo laparoscopic surgery (due to, e.g., extensive adhesion caused by prior abdominal surgery, inability to endure artificial pneumoperitoneum, etc.)
- Refusal to provide informed consent

### Sample size considerations

On the basis of previous data, the median 3-year disease-free survival (DFS) rate after CME for right-sided colon cancer is estimated to be approximately 80%, and the null hypothesis is a 3-years DFS rate after laparoscopic mCME for right-sided colon cancer of at least 88% [12, 13]. This will be assessed using an exact *p*-value of 0.025 and a power of 0.90 based on the Clopper-Pearson method. Thus, the sample size is 225. The total sample size is set to 250, considering a maximum dropout rate of about 10% for this clinical study.

### Endpoints

#### Primary endpoint

The primary endpoint is 3-year DFS, calculated as the time from surgery until the first objective documentation of recurrence or death from any cause.

#### Secondary endpoints

- The 3-year overall survival, calculated as the time from surgery until a documented death from any cause
- Incidence of postoperative morbidity until 4 weeks after surgery
- Completeness of mCME, assessed by reviewing the resected surgical specimen primarily by the pathologist at each centre and secondarily by central reviewers based on specimen photographs
- Central radicality, assessed by reviewing the operative field after specimen removal primarily by the attending surgeon at each centre and secondarily by central reviewers based on photographs obtained during the procedure
- Distribution of metastatic lymph nodes, assessed by categorising the lymph nodes retrieved from resected surgical specimens

### Participating surgeons

The participating surgeons should meet the following qualifications:

1. Has completed at least 50 cases of laparoscopic mCME in the last 3 years.
2. Has passed the blind review of surgery video. Briefly, the applicants will provide videos of laparoscopic mCME performed in the last 3 months (three cases each) to the Research Council. The Research Council will select two videos of laparoscopic mCME separately and randomly appoint three experts to perform a blind peer review. When more than two experts unanimously approve, the applicant will be permitted to participate in this study as a researcher.

### Surgical technique

Laparoscopic mCME will be carried out with endotracheal intubation under general anaesthesia. The surgeon should explore the abdominal cavity to determine if there is any distant metastasis, including hepatic, peritoneal, mesenteric, or pelvic metastasis. Thereafter, laparoscopic mCME will be performed in either a lateral-to-medial or medial-to-lateral fashion, according to the surgeon’s preference.

Similar to the original CME procedure, mCME requires the separation of the visceral fascia from the parietal fascia by sharp dissection and by ligating the supplying vessels at their origin.

Unlike the original CME, which performs consistent surgery regardless of the location of the right colon cancer, mCME has a tailored approach depending on the tumour location (Table 1).

**Table 1:** CME vs. Japanese D3 vs. mCME

	The original CME [2–4]	Japanese D3 [6, 14]	mCME [10]
**Dissection plane**	Embryologic tissue plane between retroperitoneal fascia and mesocolic fascia	Along with the layers of fusion fascia	Embryologic tissue plane respected, but obtaining secure radial margin weighed more
**Tailored lymphadenectomy**	Not mentioned	Tailored according to tumor location and stage	Tailored according to tumor location and stage
**Extra-mesocolic lymphadenectomy**	Recommended	Not recommended	Not recommended
**Securing radial margin**	Not clearly mentioned	Not clearly mentioned	Very important
**Surgical quality assessment**	Specimen grading, morphometry	Not clearly mentioned	Specimen grading as well as surgical field photo documentation after specimen removal
**Proximal & distal resection margin**	Not mentioned, but wide enough bowel resection length	10-cm rule	Bowel resection length determined by tailored lymphadenectomy

^a^ Gastroepiploic, gastrocolic trunk nodes, lymphatic tissue in pancreas capsule and other lymph nodes that are not contained in mesocolon

**Fig. 2:**
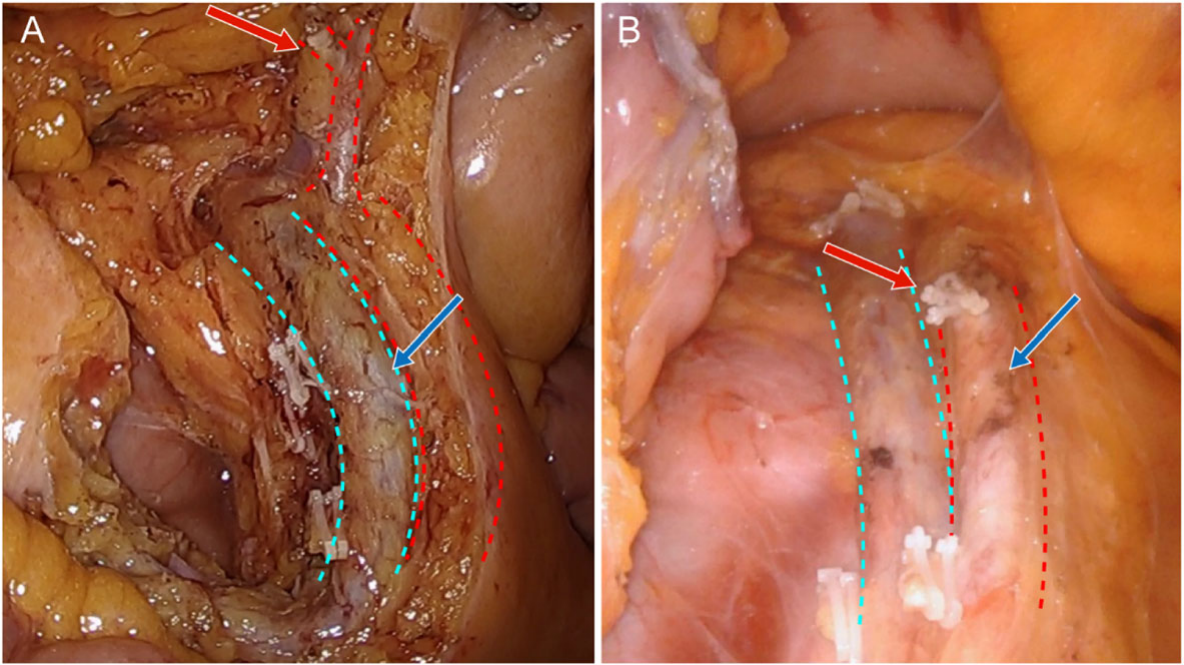
Overview of the modified complete mesocolic excision procedure with the site of the vascular ligation depending on the location of the tumour (red arrow) and the level of central radicality (blue arrow). **a** Ascending colon cancer: only the right branch of the middle colic artery is ligated. Lymphadenectomy around the origin of the colic artery with complete exposure of the superior mesenteric vein (SMV). **b** Proximal transverse colon cancer: the root of the middle colic artery is ligated. Lymphadenectomy around the origin of the colic artery with complete exposure of the SMV and the superior mesenteric artery (SMA)

First, although complete Kocherization is mandatory in the original CME description, it may be required to clear a possible tumour spread if the tumour is infiltrating or adhering to the duodenum or perinephric fat tissue. Second, if the tumour is locally advanced, the entire prerenal soft tissue behind Gerota’s fascia may need to be cleared, especially for tumours growing toward the posterior. The third difference of mCME from the conventional CME involves the tailored resection of the mesocolon and ileal mesentery according to the tumour location. After identifying the root of the middle colic artery, the site of the vascular ligation depends on the location of the tumour (Fig. 2). When the tumour is located in the caecum and ascending colon, only the right branch of the middle colic artery is ligated. If the tumour is present in these latter sites, the root of the middle colic artery is ligated. If the tumour is located in the proximal ascending colon or caecum, enough distal ileum branches of the superior mesenteric artery (SMA) are needed in the specimen to obtain a longer distal ileum. As a result, the length of the distal ileum is determined by the extent of mesenteric dissection in the mCME procedure.

### Photographic documentation

The submission of three photographs is required. We will perform a central review of the surgical procedure by evaluating the photographs obtained during the procedure for all patients. The study-specific committee will evaluate these photographs for quality control and surgical assessment, and the surgical procedure will be discussed at group meetings planned to be held twice a year. Either film or digital cameras will be used to obtain the photographs.

**Fig. 3:**
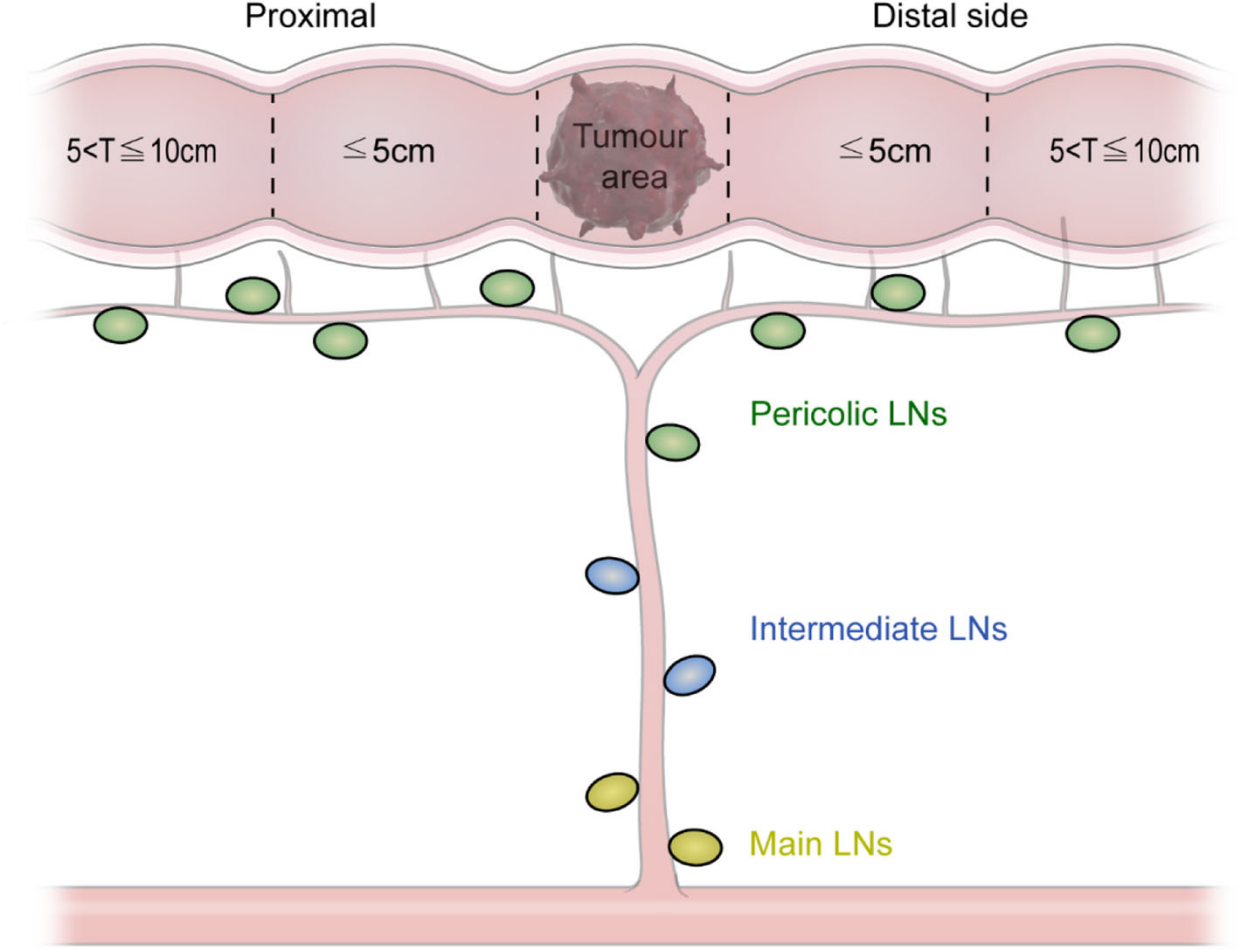
Grouping of the retrieved lymph nodes

**Fig. 4:**
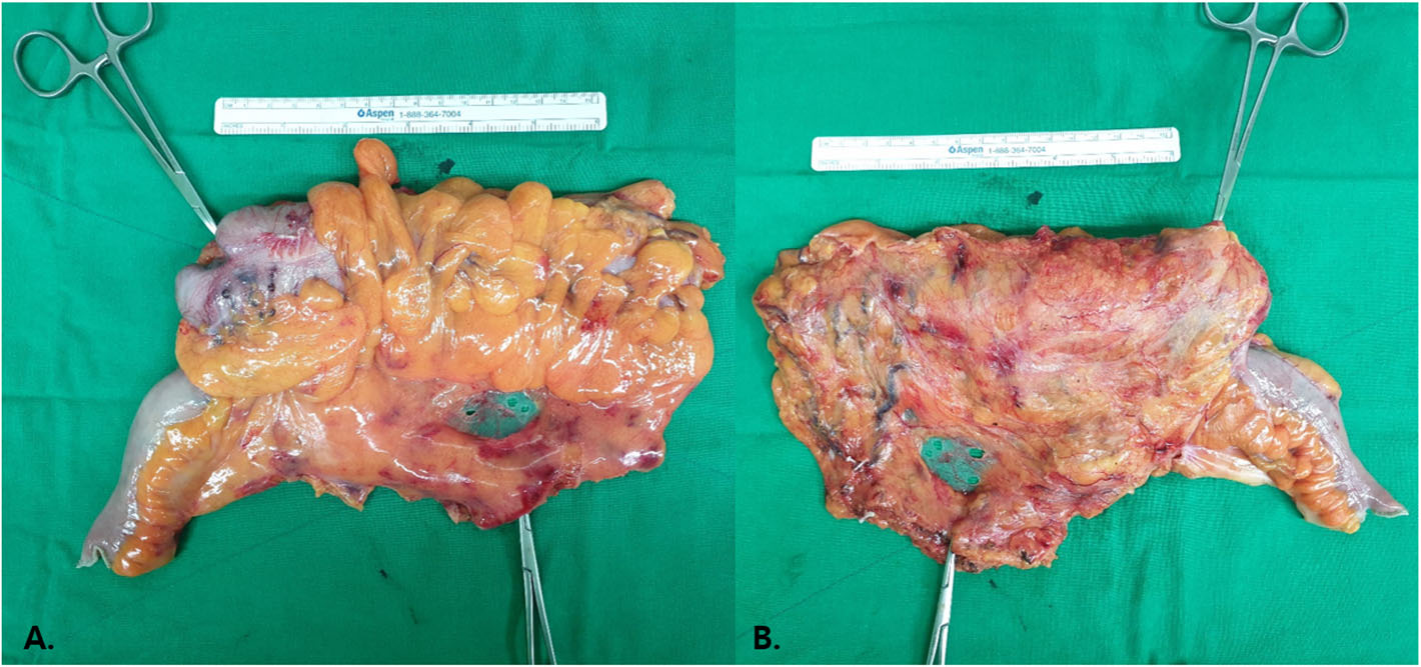
(**a**) Anterior and (**b**) posterior photographs of a fresh laparoscopic modified complete mesocolic excision specimen taken with a metric scale. The caecal tumour and ileocolic vascular tie are indicated by forceps. Note the smooth posterior surgical margin and intact peritoneal window

1. Reviewing the operative field after lymph node dissection and specimen removal based on photographs (Fig. 3)

- A photograph displaying the central radicality (extent of lymphadenectomy) will be obtained between the time after ‘lymphadenectomy and ligation of feeding arteries’ and before ‘anastomosis’.

2. Reviewing resected surgical specimens based on photographs (Fig. 4)

- High-resolution digital colour photographs of fresh specimens will be taken immediately after resection.
- Photographs of the front and back sides of the unfixed, unopened specimens, placed alongside a metric scale, will be taken.
- The mesentery must be laid out flat without stretching, and the site of the tumour and high vascular ties (HVTs) should be identifiable.

### Level of central radicality

The level of central radicality will be determined by participating surgeons based on the preoperative and intraoperative findings regarding the depth of tumour invasion and the status of lymph node metastasis.

Central radicality will be categorised into four levels according the extent of lymphadenectomy for central lymph nodes and recorded as described below (Fig. 2):

I. Lymphadenectomy around the origin of the colic artery with complete exposure of the superior mesenteric vein (SMV) and SMA
II. Lymphadenectomy around the origin of the colic artery with complete exposure of the SMV
III. Lymphadenectomy around the origin of the colic artery with partial exposure of the SMV
IV. Lymphadenectomy around the origin of the colic artery without exposure of the SMV

### Tissue morphometry

By using the photographs of the resected surgical specimens, tissue morphometry will be performed using ImageScope version 10 (Aperio, Vista, CA, USA) [4, 14]. In all cases, the mesentery will be laid out flat without stretching, and the site of the tumour and HVTs will be identifiable. The distance from the tumour and the closest bowel wall to the HVT, the length of the large and small bowel, and the area of mesentery resected will be accurately quantified.

### Macroscopic quality of the mCME specimen

The quality of mCME will be graded based on the Medical Research Council (MRC) CLASICC trial protocol depending on the plane of excision, as described below [4]. Mesocolic grading will be performed by the study committee based on a review of the submitted photographs with blinding of patient outcome. Any discrepancies between the two scores will be discussed before the final grading.

- Mesocolic plane (grade A): an intact mesocolon
- Intramesocolic plane (grade B): significant mesocolic disruptions away from the muscularis
- Muscularis propria plane (grade C): significant disruptions extending down to the muscularis

### Distribution of metastatic lymph nodes

The location of metastatic lymph nodes will be categorised into three levels based on the Japanese Classification of Colorectal Carcinoma (Fig. 3) [15].

- Pericolic lymph nodes: lymph nodes along the marginal arteries and vasa recta of the colon; those on both proximal and distal sides of the primary tumour, which are subcategorised in 5-cm intervals from the edge of the primary tumour.
- Intermediate lymph nodes: lymph nodes along the colic arteries
- Central lymph nodes: lymph nodes at the origin of each colic artery

### Adjuvant chemotherapy

Adjuvant chemotherapy will be administered based on each patient’s pathologic report and the National Comprehensive Cancer Network guideline [16].

No patient with stage I disease will plan for adjuvant chemotherapy. Patients with stage II–III disease will receive adjuvant chemotherapy using either 5-FU/leucovorin or 5-FU/oxaliplatin/leucovorin (FOLFOX) on a 2-week cycle for 12 cycles, which will be selected based on patient/physician discussions. Adjuvant chemotherapy will be recommended for patients with high-risk stage II tumours, as characterized by poorly differentiated histology (exclusive of cancers that are MSI-H), lymphovascular or perineural invasion, as well as < 12 examined lymph nodes or positive margins. FOLFOX will be recommended for patients with stage III disease, except for those in whom oxaliplatin is contraindicated due to old age, poor performance status, or pre-existing peripheral neuropathy.

### Follow-up

Patients will be scheduled for follow-up every 3 months after surgery until 3 years postoperatively. Complete blood count, blood biochemical examination (aspartate aminotransferase, alanine aminotransferase, creatinine, blood urea nitrogen, blood glucose, and cholesterol), and serum tumour marker (carcinoembryonic antigen) analyses will be performed every 3 months. Further, chest radiography, abdominopelvic computed tomography, and chest computed tomography will be performed every 6 months, and colonoscopy will be performed yearly. All results will be recorded and evaluated by a specialist.

## Discussion

High-quality surgery with the correct surgical plane improves the treatment outcomes for patients with colorectal cancer [4, 14]. Therefore, surgical quality evaluation is essential in clinical studies introducing a new surgical technique or evaluating existing procedures and is one of the key factors for success. Well-designed studies have applied strict qualification criteria for participating surgeons who have completed a learning curve for a specific procedure and make great efforts to investigate the quality of surgery as an objective indicator [4, 17]. However, it is difficult to objectively assess the quality of surgery and there are no common indicators yet.

Several studies have shown the oncological safety and feasibility of laparoscopic colon cancer surgery compared with conventional open surgery [18, 19]. However, prospective studies evaluating the safety and feasibility of laparoscopic CME, Japanese D3 dissection, and mCME in right-sided colon cancer surgery are rare. Furthermore, there are few studies involving quality assessments of right-sided colon cancer surgery using objective measures, and there have not been any definitive clinical studies evaluating surgical quality using intraoperative photographs in laparoscopic right-sided colon cancer surgery.

The PIONEER study is a multi-institutional, prospective, single-arm study evaluating oncologic outcomes after laparoscopic mCME for adenocarcinoma arising from the right side of the colon (defined as from the caecum up to the proximal half of the transverse colon). Remarkably, the study will also evaluate the quality of surgery with objective and rigorous indicators of laparoscopic mCME performed by certified surgeons, which will ultimately demonstrate the significance of surgical quality in treatment outcomes.

Notable previous randomised controlled trials comparing open and laparoscopic colorectal surgery confined the criterion for participating surgeons to performing at least 20 laparoscopic resections [17–19]. However, in this study, only surgeons who performed at least 50 cases of laparoscopic mCME were qualified to participate; therefore, a more stringent criterion was applied here as compared to other previous prospective studies. Moreover, this study adopts a dual-valuation system based on photographs of the surgical field taken during the operation and those of the resected specimens, to maintain the surgical quality and to improve the objectivity of evaluation. For surgeons participating in the PIONEER study, submitting three photographs is mandatory. Photographs to be submitted include a photograph of lymph node dissection and photographs of the front and back sides of the resected surgical specimen for surgical quality control and assurance.

The completeness of mCME will be graded based on the MRC CLASICC trial protocol, by reviewing the photographs of the resected surgical specimen primarily by a pathologist at each centre and finally by central reviewers [4]. The mCME procedure follows the salient concept of the original CME technique (oncologic resection with careful dissection of the mesocolon along the embryologic planes resulting in the complete mobilisation of the mesocolon covered by an intact visceral fascia layer containing all blood vessels, lymphatic vessels, and lymph nodes that may contain disseminated cancer cells) but with a more tailored approach [2, 10]. Therefore, evaluating the smoothness of the fascia covering the resected mesocolon is considered a powerful method for assessing the completeness of mCME, which is a good prognostic factor for colon cancer [4, 14]. However, the weakness of this grading system is that even if the peritoneal-lined mesentery and fascia covering the mesocolon are intact, it does not assess the tumour involvement of the radial margin, which is a risk factor for recurrence [20]. The mCME has different features from the original CME, one of which is securing an adequate radial margin [10]. Consequently, this study is expected to achieve a clearer evaluation of the quality of laparoscopic right-sided colon cancer surgery by overcoming the weakness of the mesocolic grading system.

Precise quantitation of the resected surgical specimen, by measuring the total area of the resected mesocolon and the distance from the muscularis propria to the nearest mesenteric and radial margin, is an indicator of surgical and oncological quality, as well as the measuring plane of surgery and lymph node retrieval [14]. However, considering the surgical principle that central ligation of main supplying vessels reduces the risk of residual metastatic lymph nodes and enables accurate staging, it is very challenging to assess the extent of lymph node dissection and CVL by using tissue morphometry and the number of retrieved lymph nodes. To assess the central radicality of laparoscopic mCME, the surgical field after lymph node dissection and specimen removal will be reviewed primarily by the attending surgeon at each centre and finally by central reviewers based on the photographs obtained during the procedure.

To our knowledge, this study will be the first prospective trial to assess the oncologic safety of laparoscopic mCME for right-sided colon cancer, supported by the thorough surgical quality control system of the study group. Patient-tailored surgical treatment for right-sided colon cancer can be developed based on the present study results, including the determination of the optimal extent of dissection and the extent of the lymphadenectomy. Additionally, it may aid in the development of a global standardised surgical procedure for right-sided colon cancer.

## Data Availability

Not applicable in this study protocol. After finishing the enrollments of the participants, the raw datasets are available from the corresponding author on reasonable request.

## Abbreviations

CVL: central vascular ligation
CME: complete mesocolic excision
DFS: disease-free survival
HVTs: high vascular ties
MRC: Medical Research Council
mCME: modified CME
SMA: superior mesenteric artery
SMV: superior mesenteric vein
